# Tuberculosis-Related Deaths within a Well-Functioning DOTS Control Program

**DOI:** 10.3201/eid0811.020021

**Published:** 2002-11

**Authors:** Maria de Lourdes García-García, Alfredo Ponce-de-León, Maria Cecilia García-Sancho, Leticia Ferreyra-Reyes, Manuel Palacios-Martínez, Javier Fuentes, Midori Kato-Maeda, Miriam Bobadilla, Peter Small, José Sifuentes-Osornio

**Affiliations:** * Instituto Nacional de Salud Pública, Cuernavaca, México; † Instituto Nacional de Ciencias Médicas y Nutrición “Salvador Zubirán,” México City, México; ‡ Instituto Nacional de Enfermedades Respiratorias, México City, Mexico; § Secretaría de Salud del Estado de Veracruz, Xalapa, México; ¶ Stanford University, Palo Alto, California, USA

## Abstract

To describe the molecular epidemiology of tuberculosis (TB)-related deaths in a well-managed program in a low-HIV area, we analyzed data from a cohort of 454 pulmonary TB patients recruited between March 1995 and October 2000 in southern Mexico. Patients who were sputum acid-fast bacillus smear positive underwent clinical and mycobacteriologic evaluation (isolation, identification, drug-susceptibility testing, and IS6110-based genotyping and spoligotyping) and received treatment from the local directly observed treatment strategy (DOTS) program. After an average of 2.3 years of follow-up, death was higher for clustered cases (28.6 vs. 7%, p=0.01). Cox analysis revealed that TB-related mortality hazard ratios included treatment default (8.9), multidrug resistance (5.7), recently transmitted TB (4.1), weight loss (3.9), and having less than 6 years of formal education (2). In this community, TB is associated with high mortality rates.

In both humanistic and economic terms, the cost of deaths due to tuberculosis (TB) is staggering. In 1990 alone, approximately 2.5 million people died of TB, accounting for >25% of avoidable adult deaths in the developing world (1,2). Directly observed treatment strategy (DOTS), a comprehensive approach to TB control, is one of the most cost-effective health interventions available (3,4). In the context of a well functioning DOTS program, cure rates in excess of 80% can be expected. While these outcomes are assumed to decrease mortality rates, the detailed epidemiology of deaths in a well-functioning DOTS program by using modern molecular techniques has not been described.

Since 1995, we have conducted a population-based molecular epidemiologic study of TB in a health district in southern Mexico. Previous reports have documented the TB control program approaches World Health Organization benchmarks (5) and drug resistance is considerable and has an important negative impact on treatment outcomes (6). We now report the short- and long- term mortality rates due to TB in this cohort of TB patients. These data suggest that, as has been described with other diseases, excess mortality may persist for months or years after treatment completion, default, or failure (7,8).

## Methods

The study site, described previously (5,6,9), is located in a predominantly urban region in the Orizaba Health Jurisdiction in the state of Veracruz, which encompasses 134 square km and has a population of 284,728 (10). The incidence rate of TB during the year 2000 for the state was higher than that for the nation (28.0 vs. 15.9 per 100,000 inhabitants) (11).

Community-based screening of chronic coughers (>2 weeks) was performed from March 1995 to October 2000. Patients with positive AFB sputum smears underwent epidemiologic, clinical (standardized questionnaire, physical exam, chest x-ray, and HIV test), and mycobacteriologic evaluation. Treatment was provided in accordance with official norms (12,13). Treatment outcomes were classified as previously described (6). Annual follow-up was performed for treatment outcome and vital status. Deaths were confirmed with death certificates. A close caregiver was interviewed to elicit signs and symptoms of the terminal illness and “probable cause of death” was assigned by two of the authors (JF, LF). Informed consent was obtained from participants. The study was approved by the appropriate institutional review boards.

### Microbiologic Evaluation

Mycobacterial culture, identification, and susceptibility testing were performed on sputa from each enrolled patient. In brief, unconcentrated sputum was spread onto Lowenstein-Jensen media (DIFCO, Detroit, MI) at the local laboratory, and the remaining sputum was frozen at –70°C. The tubes were examined on a weekly basis until growth was detected. Cultures were reported as negative if no growth occurred after 8 weeks. Cultures with visible growth were forwarded to the department of Mycobacteriology at the Instituto de Diagnostico y Referencia Epidemiológicos (March 1995 to December 1997) or to the Clinical Microbiology Laboratory of the Instituto Nacional de Ciencias Médicas y Nutrición (INCMNSZ) (January 1998 to October 2000) for definitive biochemical identification at the species level (14,15). The frozen sputum sample was processed if the first sample was contaminated or had no growth. Identification and drug susceptibility tests were carried out using conventional methods and the BACTEC system (Becton Dickinson, Cockeysville, MD).

### Mycobacterium tuberculosis Fingerprinting

Mycobacteria isolated from study patients were genotyped at Stanford University from March 1995 to February 1997 and at INCMNSZ from March 1997 to October 2000 with the internationally standardized IS6110-based restriction fragment length polymorphism (RFLP) technique and compared by using a computer-assisted visual approach (Bioimage AQ-1 analyzer and Molecular Fingerprinting Analyzer, version 2.0) (16,17). Isolates with identical IS6110 fingerprints that contained five or fewer hybridizing bands underwent spoligotyping at INCMNSZ as described (18,19). To assess transmission of M tuberculosis that rapidly progressed to disease, we established a 1-year period for defining clustering as described (20). Cases were considered “clustered” if two or more isolates from different patients were identified within a year that had 1) six or more IS6110 bands in an identical pattern, or 2) five or fewer bands with identical IS6110 fingerprints and matching spoligotypes. The first patient diagnosed in each cluster and those with unique fingerprints were classified as having reactivated disease.

## Analysis

Deaths were attributed to TB based on the death certificate (TB listed as the cause of death), interview of close caregiver (TB identified as probable cause of death), and active TB at the time of death (positive AFB smear after last treatment or during treatment if the patient did not complete treatment performed <6 months before death). A patient’s death was attributed to TB if two or more of these conditions were met. Bivariate and multivariate analyses were performed to describe association between sociodemographic (age, sex, household characteristics, occupation, ethnicity, years of formal education, place of residence, and social security), behavioral (usage of drugs and alcohol, and previous incarceration), clinical (previous diagnosis of TB; other associated diseases, previous hospitalizations; HIV infection, duration of symptoms previous to diagnosis; time elapsed between diagnosis and initiation of treatment and between initiation of treatment and smear conversion; and symptoms such as cough, hemoptysis, fever, night sweats, weight loss, and general malaise), bacteriologic (number of bacilli per microscopic field, drug resistance, RFLP, or spoligotype pattern), therapeutic (compliance, treatment outcome, and retreatment) variables, and death due to TB and other causes. Survival analyses included Kaplan-Meier curves and Cox proportional hazards model for TB and non-TB–related deaths using as reference time the period elapsed from diagnosis. Variables were entered into the models according to their statistical significance in univariate analysis and their biological relevance. The percentage of population attributable risk was calculated for variables that were included in the final model (21). DBASE IV and STATA 5.0 programs were used for data analysis (22).

## Results

During the study period, 454 patients were diagnosed with pulmonary TB. Most patients were men (270 [59.5%] of 454) and the median age was 42 years (range 12–97 years). Most came from lower socioeconomic status as indicated by household characteristics, formal years of education, and occupation. Of 438 patients, 74 (17%) lived in households with earthen floor, 214 (49.3%) of 434 had no access to potable water within the household, 293 (65.4%) of 448 had <6 years of formal education, and 104 (23.4%) of 445 were manual workers. The prevalence of HIV was 2.1% (9 of 429), combined resistance to isoniazid and rifampin was seen in 26 (6.7%) of 388 patients, and other resistance patterns in an additional 61 (15.7%) of 388. One hundred fifty-one (37%) of 413 patients had chest radiographs with evidence of pulmonary cavitation, and 72 (17.4%) of 413 patients had interstitial pulmonary infiltrates. Median time from initiation of symptoms to treatment was 101 days (range 3–2,307 days), median time from diagnosis to initiation of treatment was 5 days (range 0–594 days), and median time for sputum conversion was 42 days (range 15–1,228 days).

### Treatment and Follow-Up

Of 11 patients who refused treatment, 4 died. Of 443 initiating treatment, 75.4% initiated treatment <10 days after diagnosis; 96.5% received supervised treatment. Outcomes for patients were as follows: 357 (80.6%) were cured of whom 314 (70.9%) were bacteriologically confirmed, 41 (9.3%) defaulted, and 20 (4.5%) failed treatment; 16 (3.6%) died during treatment, and 9 (2%) transferred out of study area. Patients were tracked for a median of 839 days (range 3–2,402). Sixty-one additional patients died during follow-up after treatment. Death was due to TB in 34 (41.9%) of 81 instances; 2 deaths were in patients who did not receive treatment, 11 in patients receiving treatment, and 21 after treatment. Tuberculosis mortality rates were higher during treatment versus after treatment (1.3/10,000 days vs. 0.7/10,000 days, p<0.01). Crude comparison of sociodemographic, bacteriologic, and clinical characteristics of patients who died from TB or from other causes and surviving patients are shown in Table 1. Patients who died from TB had a higher probability of having been treated for a previous TB episode, showed more severe clinical symptoms, and had drug-resistant isolates. Less frequently they had other coexisting chronic conditions, such as HIV infection or hepatic cirrhosis. The patients who died from TB also had longer delays before diagnosis, treatment, and sputum conversion. These patients had higher probabilities of default, failure, and having subsequent TB episodes. The most common non-TB causes of death included diabetes (12), cirrhosis and other liver diseases (9), AIDS (6), cancer (2), and cardiovascular diseases (3). Kaplan Meier survival probabilities from TB deaths were 97.3% after the first 6 months, 95.8% after 1 year, 93.7% after 2 years, and 91% after 3 years.

**Table 1:** Sociodemographic, clinical, bacteriologic, and therapeutic characteristics of smear-positive pulmonary tuberculosis (TB) patients according to cause of death, Orizaba, Veracruz, 1995–2000

Variables	Died from TB (n=34) (%)	Died from other causes (n=47) (%)	Survived (n=373) (%)	p valuea
Sociodemographic				
Median age (range)	30 (24–73)	47 (22–70)	40.5 (12–82)	0.05
Men	52.9	76.6	57.9	0.04
Indigenous origin	20.6	4.3	16.1	0.07
<6 years formal education	73.5	76.6	62.2	0.02
Rural and industrial workers	11.8	25.5	23.6	0.2
Previous imprisonment	14.7	34.0	27.9	0.2
Previous TB treatment	47.1	34.0	14.2	<0.0001
Previous hospitalization	58.8	51.1	45.6	0.2
Residence in shelters	5.9	10.6	5.1	0.3
Alcohol use	38.2	66.0	43.7	0.005
Household crowding	47.1	27.7	37.5	0.2
Household with earthen floor	20.6	10.6	16.6	0.4
Clinical				
HIV infection	8.8	10.6	.3	<0.0001
Hepatic cirrhosis	0	6.4	1.3	0.06
Body mass index (<18)	47.1	27.7	20.4	<0.0001
Hemoptysis	26.5	38.3	37.8	0.5
Fever	44.1	61.7	44	0.04
Night sweats	58.8	59.6	57.6	0.2
Weight loss (>15 %)	47.1	46.8	29.2	0.002
Radiologic nodes	5.9	6.4	7.0	0.9
Cavities	44.1	25.5	33.2	0.2
Median time interval between initiation of symptoms and treatment (range in days)	17 (1–212)	8 (0–158)	5 (0–322)	0.004
Median time interval between diagnosis and treatment (range in days)	141.5 (78–991)	126 (4–439)	99.5 (4–1,723)	0.01
Bacteriologic				
Resistance to isoniazid and rifampin	29.4	17.0	2.1	<0.0001
Other resistance	11.8	19.1	12.9	0.2
<10 bacilli per 100 fields	79.4	87.2	86.1	0.5
Median time interval between treatment and sputum conversion (range in days)	95 (35–530)	44 (19–182)	42.5 (15–348)	0.03
Treatment outcome				
Cure	5.9	61.7	87.4	<0.0001
Failure	20.6	10.6	2.1	<0.0001
Default	32.4	10.6	6.7	<0.0001
Retreatment	17.6	23.4	5.4	<0.0001

aChi square test, analysis of variance test.

### RFLP and Spoligotyping Results

M. tuberculosis culture, drug test, and IS6110 RFLP and spoligotyping were available for 326 (72%) isolates. Comparison of patients whose isolates were available for genotyping to those whose isolates were unavailable indicated that patients for whom fingerprint analysis was not performed had a higher probability of being of native origin in Mexico (30 [24.2%] of 124 vs. 39 [12%] of 324, p= 0.001) and of living in households with earthen floor (31 [25.8%] of 120 vs. 43 [13.5%] of 318, p=0.002). Forty (12.3%) of the 326 evaluated cases were in clusters. The frequency of being members of clusters of recently transmitted disease was higher among patients dying from TB than among those dying from other causes or surviving (8 [28.5%] of 28 patients who died from TB vs. 32 [10.7%] of 298 patients who died from other causes and survivors, p=0.01).

### Factors Associated with Mortality Rates

Predictors of death due to TB by Cox regression analysis included treatment default, resistance to isoniazid and rifampin, and recently transmitted TB controlling for time of occurrence of death, weight loss >15%, and years of formal education (Table 2). The effect was not modified when gender, age, HIV infection, crowding in the household, household characteristics, occupation, ethnicity, previous treatment, delay in seeking treatment, specific symptoms, type of radiologic lesions, and other kinds of diseases were introduced into the model.

**Table 2:** Population attributable-risk percent and hazard ratios for death among smear-positive tuberculosis (TB) patients, Orizaba, Veracruz, 1995–2000a

Variables	Population attributable risk (%)	Adjusted hazard ratio	95% CI	p valueb
Death due to TBc				
Treatment default	28.7	8.9	3.3 to 24.4	<0.0001
Resistance to isoniazid and rifampin	25.9	5.7	2.0 to 16.3	<0.001
Clustered	18.8	4.1	1.6 to 10.0	0.002
Weight loss (>15%)	—	3.9	1.5 to 10.9	0.007
Formal education <6 yr	—	1.8	0.6 to 5.2	0.3
Death due to other causes				
HIV/AIDS	11.1	33.1	11.4 to 95.4	<0.0001
Hepatic cirrhosis	6.6	5.7	1.6 to 19.7	0.006
Weight loss (>15% )	—	3.3	1.6 to 6.7	0.001
Age (yrs)	—	1.02	0.99 to 1.04	0.07

aCI, confidence interval; —, not applicable. bCox proportional hazards model. cControlling for death before or after treatment completion, default or failure.

After controlling for age, predictors of non-TB death included HIV-infection, hepatic cirrhosis, and weight loss. Recently transmitted TB was not associated with other causes of death (Table 2).

The proportion of death due to TB and to other causes attributable to the different categories of risk factors is shown in Table 2. In the study population, 60% of deaths due to TB were attributable to drug resistance and treatment default.

Patients with recently transmitted disease had a lower probability of survival compared with patients with reactivated disease (p=0.007) (Figure). When causes of death were analyzed according to genotype, we found that TB was the cause of death in 8 (20%) of 40 patients with recently transmitted disease and in 20 (7%) of 286 patients with reactivated disease (p=0.01).

**Figure:**
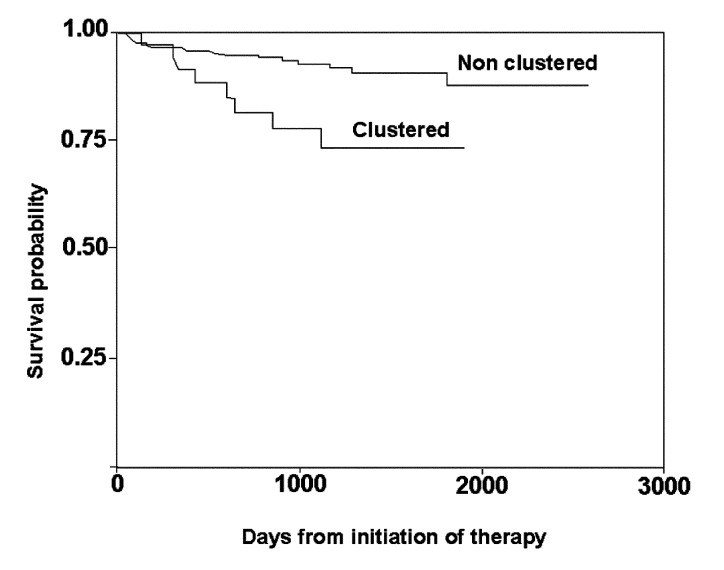
Estimated survival of smear-positive pulmonary tuberculosis patients according to clustered or unique fingerprint pattern in a low HIV-prevalence community (p=0.01).

### Sensitivity Analysis

Analysis of the distribution of the time between diagnosis dates of successive matching fingerprints indicated that 49.4% (95% confidence interval [CI] 44% to 54%) of all isolates with matching fingerprints patterns were identified within 1 year. When the interval was modified to 6, 18, 24, 30, and 36 months, we found that the proportion of clustered cases increased (8.3%, 13.8%, 13.8%, 14.4%, and 14.7%, respectively), (χ2 trend, p=0.02). The association between clustered cases and death due to TB continued to be positive for each of the other definitions of interval for clustering: 3.6 (1.1 to 10.5), p=0.01; 2.8 (1.0 to 7.3), p=0.03; 2.8 (1.0 to 7.3), p=0.03; 2.7 (0.9 to 6.8), p=0.04; and 2.6 (0.9 to 6.6) p=0.04.

## Discussion

This study describes high mortality rates from TB in a cohort of pulmonary TB patients who resided in an area with a low rate of HIV infection and were treated in the context of a well-functioning DOTS program. Patients were followed for an average of 2.3 years after diagnosis. Tuberculosis was associated with high rates of deaths both during treatment and after treatment completion, default, or failure. The main independent risk factors for death due to TB were treatment default and being infected with multidrug-resistant M. tuberculosis. Additionally, data indicate that cases due to ongoing transmission of TB may have higher mortality rates than cases due to reactivation of latent disease. These results suggest that current techniques underestimate death associated with TB and provide further impetus not only to treat but also to prevent TB.

Case completion rates are the standard by which the effectiveness of DOTS-based treatment programs are judged. A large body of evidence collected under diverse settings demonstrates completion rates of 81% and cure rates of 73% (23). The epidemiology of death in these programs is less well studied. Studies performed in HIV-endemic settings constitute an exception as the high rate of deaths occurring while patients are still on antituberculous therapy are cited as an impediment to achieving desired cure rates (24).

Our study reports TB mortality rates both during and after treatment completion, default, or failure. Of the 443 patients who were started on therapy, 16 died before completing therapy. This frequency (3.6%) compares favorably to that reported in other studies (4% in the Gambia [25], 15% in Bolivia [26], 25% in Malawi [27], and 28% in India [28]). However, in contrast to previous reports, we had a median of 833 days of follow-up after the completion of therapy, during which 21 additional patients (4.6%) died of TB, comparable with reports from the 1960s and 1970s (29). This mortality rate exceeds that expected according to age-adjusted state mortality statistics (30).

Although not novel, the risk factors for death due to TB identified in this cohort are noteworthy; these findings suggest that, even in a high-quality TB control program, additional efforts could yield important benefits. Treatment default has been previously described as associated with higher mortality rates in Mexico (31) and elsewhere (32,33). Concerted efforts to further reduce default may disproportionately decrease deaths. We also found that drug resistance, and particularly multidrug resistance, are associated with death (6,14,34). Although the most appropriate strategy for managing drug-resistant TB is debated, these data provide additional impetus for evaluating novel approaches, such as those recently introduced in Mexico, for managing drug-resistant TB (13,35). We estimate that 60% of deaths due to TB in this setting were attributable to treatment default and multidrug-resistance. Additional studies are needed to quantitate the presumably greater mortality rates that result from default and drug resistance in other settings.

Although relatively uncommon in this setting, HIV was associated with a high overall mortality rate. Most of these deaths were due to non-TB–related death. HIV/AIDS was the main predictor for non-TB deaths in this cohort. In our study, eight of the nine HIV-seropositive persons died, three of them a year or more after completing antituberculous therapy. Several studies have demonstrated excess mortality rates after successful TB treatment in HIV-infected patients (24,36,37); the high rates have been attributed to HIV-related disease (38). These data emphasize the need to improve integration of quality treatment for both TB- and HIV-infected persons in populations that suffer from both diseases.

The most striking finding of the molecular epidemiologic component of this study is the association between clustering, which we interpret as indicative of recently transmitted disease, and TB-related death. Death due to TB was significantly more common among those who had recently transmitted disease than those with reactivated disease (28.6% vs. 7%, p=0.01). This association was independent of treatment default, multidrug resistance, time of occurrence of death, weight loss, and years of formal education. Furthermore, the effect was not modified when other variables indicative of sociodemographic level (such as occupation, characteristics of the household, or ethnicity) or clinical variables indicating other diseases, including HIV infection were introduced in the model. Inferring causality from such associations is difficult, it seems biologically implausible that being more likely to die made people more likely to acquire recently transmitted TB. A more plausible explanation is that rapidly progressing to disease contributes to deterioration of the health of these patients and thus increases their likelihood of death. The phenomenon is well described for other diseases such as measles. However, why this would be more pronounced for recently transmitted disease is unclear.

The association between clustering and death could be spurious because of limitations in molecular or conventional epidemiology. The validity of clustering as a proxy for recent transmission might be challenged by the fact that we did not perform fingerprint analysis on all isolates (39). Comparison between patients whose isolates were genotyped and those whose isolates were not genotyped showed that results of this study may not be generalizable to indigenous or lower socioeconomic groups. Our selection of the time interval allowed us to consider TB that was transmitted very recently and progressed to disease. The validity of using a 1-year interval was confirmed with the sensitivity analysis using different time periods (6, 18, 24, 30, and 36 months) as association between clustered cases and death persisted, despite modification of the time interval and by the fact that almost 50% of isolates with matching DNA fingerprint patterns occurred within 1 year of identification of the previous case. Conventional epidemiologic approaches to studying the cause of death are difficult. Death certificate data are notoriously unreliable, and whether patients died of TB or other causes is not certain (40). Therefore, we added other criteria that included the interview of a close caregiver and activity of TB at the time of death to validate our definition of death due to TB. The cause-of-death profile derived from interview of a close caregiver has been demonstrated to be useful for planning purposes (41). We consider that this definition adequately identified TB-related death as characteristics of patients dying from TB differed from patients dying from other causes in several important aspects (drug resistance, coexisting chronic conditions, and clinical severity) and allowed the identification of different risk factors for TB-related death and for death due to other causes.

If confirmed in other settings, the conclusions of this study have important implications for control programs. Most importantly, given that current surveillance data are collected at the conclusion of therapy, this method probably underestimates the true impact of TB on a population’s death. Given the increasing role of cost-efficacy modeling in setting health-care priorities, this oversight has important consequences. In addition, if recently acquired TB exhorts a greater mortality rate than that due to reactivated infection, the importance of interrupting TB transmission is further elevated.
